# Psychometric evaluation of spinal assessment methods to screen for scoliosis in children and adolescents with cerebral palsy

**DOI:** 10.1186/s12891-015-0801-1

**Published:** 2015-11-14

**Authors:** Måns Persson-Bunke, Tomasz Czuba, Gunnar Hägglund, Elisabet Rodby-Bousquet

**Affiliations:** Department of Clinical Sciences, Lund, Orthopaedics, Lund University, S-221 85 Lund, Sweden; National Competence Center for Quality Registers, Lund University, University Hospital, S-221 85 Lund, Sweden; Centre for Clinical Research, Uppsala University, County Hospital, S-721 89 Västerås, Sweden; Department of Orthopaedic, Skane University Hospital, SE 221 85 Lund, Sweden

**Keywords:** Cerebral palsy, Scoliosis, Reliability, Validity, Examination, Screening, Measurement, Neuromuscular scoliosis, Cobb angle, Psychometric evaluation

## Abstract

**Background:**

In cerebral palsy (CP) there is an increased risk of scoliosis. It is important to identify a progressive scoliosis early-on because the results of surgery depend on the magnitude of the curve. The Swedish follow-up program for cerebral palsy (CPUP) includes clinical examinations of the spine. The reliability and validity of the assessment method have not been studied. In this study we evaluate the interrater reliability of the clinical spinal examination used in CPUP and scoliometer measurement in children with CP and we evaluate their validity compared to radiographic examination.

**Methods:**

Twenty-eight children (6–16 years) with CP in Gross Motor Function Classification System levels II-V were included. Clinical spinal examinations and scoliometer measurements in sitting position were performed by three independent examiners. The results were compared to the Cobb angle as determined by radiographic measurement. Interrater reliability was calculated using weighted kappa. Concurrent validity was analyzed using the Cobb angle as gold standard. Sensitivity, specificity, area under receiver operating characteristic curves (AUC) and likelihood ratios (LR) were calculated. Cut-off values for scoliosis were set to ≥20° Cobb angle and ≥7° scoliometer angle.

**Results:**

There was an excellent interrater reliability for both clinical examination (weighted kappa = 0.96) and scoliometer measurement (weighted kappa = 0.86). The clinical examination showed a sensitivity of 75 % (95 % CI: 19.4–99.4 %), specificity of 95.8 % (95 % CI: 78.9–99.9 %) and an AUC of 0.85 (95 % CI: 0.61–1.00). The positive LR was 18 and the negative LR was 0.3. The scoliometer measurement showed a sensitivity of 50 % (95 % CI: 6.8–93.2 %), specificity of 91.7 % (95 % CI: 73.0–99.0 %) and AUC of 0.71 (95 % CI: 0.42–0.99). The positive LR was 6 and the negative LR was 0.5.

**Conclusion:**

The psychometric evaluation of the clinical examination showed an excellent interrater reliability and a high concurrent validity compared to the Cobb angle. The findings should be interpreted cautiously until research with larger samples may further quantify the psychometric properties. Clinical spinal examinations seem appropriate as a screening tool to identify scoliosis in children with CP.

## Background

Children and adolescents with cerebral palsy (CP) have an increased risk of scoliosis [1]. The reported prevalence varies between 15–64 % based on age, severity of CP, and different definitions of scoliosis [1–3]. This stands in contrast to idiopathic scoliosis in adolescents where the prevalence has been reported at 2–4 % [4]. In children with CP the risk of developing scoliosis is related to the child’s gross motor function and age [1, 5]. Severe scoliosis is associated with pain, sitting problems, hip dislocation, and windswept deformity [1, 6], all of which may impair physical function and quality of life.

It is important to identify a progressive scoliosis early-on as the result of spinal surgery is related to the curve magnitude [7]. It is desirable to have a screening tool with high sensitivity but it is also important to have high specificity to avoid unnecessary radiographic examinations.

In 1994, a follow-up program and registry for children and adolescents with CP (CPUP) was initiated in the south of Sweden, an area of approximately 1.3 million inhabitants. CPUP has been classified as a Swedish National Health Care Quality Registry since 2005 and the program is also used in Norway, Denmark, Iceland, Scotland and New South Wales, Australia. Currently >95 % of all children with CP in Sweden participate in CPUP [8] and it is expanding to include also adults with CP.

The main purpose of CPUP is to prevent hip dislocation, contractures, scoliosis and windswept deformities in individuals with CP [8–11]. The program includes spinal examinations where the children undergo a standardized examination by their local physiotherapist twice a year until six years of age and then once a year. The spine is examined in forward bending and upright position with the child sitting on a plinth. In the event of scoliosis, it is graded as “mild”, “moderate” or “severe”. A child with a moderate or severe scoliosis is referred to radiographic examination. If the Cobb angle exceeds 40° operative treatment is considered.

A commonly used screening-tool for adolescent idiopathic scoliosis is the forward bending test that measures asymmetrical rib prominence [12, 13]. It has been argued that this test does not have a quantitative documentation and the efficacy of the test to screen for scoliosis is still discussed [12, 13].

The scoliometer [14] reliably measures the angle of trunk rotation and it is a commonly used tool to screen for adolescent idiopathic scoliosis [13, 15]. The recommended cut-off value to warrant a radiographic referral in adolescent idiopathic scoliosis varies but ≥7° has been suggested [15]. To our knowledge the scoliometer has not been used in screening for neuromuscular scoliosis.

The Cobb angle has been the gold standard to quantify scoliosis on radiographic examination since 1948 [16]. However, the Cobb angle is a two-dimensional analysis of a lateral deviation of the spine while the forward bending test and the scoliometer reflect a lateral deviation and rotation of a three dimensional deformity. Coehlo et al. found a correlation that was considered good (r > 0.7, *p <* 0.05) between scoliometer measurement and the Cobb angle in screening for adolescent idiopathic scoliosis [17].

For a screening tool to be useful in clinical practice a high sensitivity and a high specificity is important. When screening for scoliosis in CPUP the purpose is to identify all individuals requiring further radiographic examination and rule out those who do not in order to minimize the dose of radiation exposure.

The purposes of this study were to evaluate the interrater reliability of clinical examination and scoliometer measurement, and to evaluate their sensitivity, specificity and concurrent validity as screening tools by using radiographic examination with the Cobb angle as gold standard.

## Methods

In CPUP, all participating children have their CP diagnosis verified by a neuropaediatrician at four years of age. CP is defined as a non-progressive brain injury which has developed before the age of two years. Motor impairment and specific neurological signs are defined and classified according to the inclusion criteria of the Surveillance of Cerebral palsy in Europe (SCPE) network [18, 19]. Gross motor function is determined by the child’s physiotherapist according to the expanded and revised version of the Gross Motor Function Classification System (GMFCS) [20, 21]. This is a 5-level system for children and adolescents with CP based on their self-initiated movement where level I delineates the highest level of function and level V the lowest.

Children and adolescents aged 6–16 years at GMFCS levels II-V and enrolled in CPUP were recruited from five child rehabilitation units in southern Sweden. The participants and their families were informed about the study by their local physiotherapists and provided with invitation letters with information about the study. Written consent was obtained from the parents/guardians of all participants. Children were recruited consecutively until at least six children at each relevant GMFCS level had accepted. The decision to include six persons in each GMFCS level (except level I) was based on an earlier study evaluating the Posture and Postural Ability Scale in adults with CP [22, 23]. In addition, a reliability study of the scoliometer by Bonagamba and colleagues included 24 participants and that study had enough power to satisfactorily evaluate reliability [24]. Children at GMFCS level I, which constitutes about 40 % of all children with CP, do not have a higher risk of scoliosis compared to the risk of developing idiopathic scoliosis in adolescents [1] and were therefore not included in this study.

The children were examined at one occasion by three examiners, independent of each other, during a period from November 2013 to March 2014. The examinations were performed by two physiotherapists and one paediatric orthopaedic surgeon, all with 10 to 20 years of experience working with children with CP. Each examiner assessed each child individually and noted the grading on separate scoring sheets. They had no information of the children’s medical reports or radiographs, and they had not met the children prior to the examinations. All three examiners had previous experience of clinical examinations of children with CP, but only the orthopaedic surgeon had experience of scoliometer measurements in adolescent idiopathic scoliosis prior to this study. The spine was examined with the child in a sitting upright position, with external support if needed, and then, still in sitting, with the forward bending. The degree of scoliosis was noted according to the CPUP classification [1] and graded as:

- *No* scoliosis.
- *Mild scoliosis*: discreet curve visible only on thorough examination in forward bending.
- *Moderate scoliosis*: obvious curve in both upright and forward bending.
- *Severe scoliosis*: pronounced curve preventing upright position without external support.

In sitting position, a scoliometer was placed in forward bending at the top of the thoracic spine, with the 0 (zero) mark over the spinous process, and slowly moved down the spine noting the highest degree of truncal rotation. The degree of scoliosis was recorded separately and independently by the three examiners. A higher degree of truncal rotation indicates worse inclination. The value used to detect moderate scoliosis that should be referred to radiographic examination was set to ≥7°.

Radiographic examinations were performed in a sitting position, in an anteposterior (AP) projection. The magnitude of the curve was determined based on the Cobb angle [16] and moderate or severe scoliosis was defined as Cobb angle ≥20°. The radiographic examinations were performed at three radiology departments using standardized instructions for patient position and x-ray imaging. The Cobb angles were measured by three different radiologists with a special interest in skeletal radiology. The measurements were reevaluated and confirmed by a fourth independent examiner. None of the radiologists had information about the previous results of the clinical examinations or the scoliometer measurements.

### Statistical analyses

In the statistical analysis the interrater reliability for the clinical spinal examination and the scoliometer measurement was calculated using weighted kappa scores [25]*.* The magnitude of weighted kappa was interpreted according to Fleiss 1981 where <0.40 signifies poor agreement, 0.40–0.74 fair to good agreement and *≥*0.75 signifies excellent agreement [26]. To calculate 95 % CI for weighted kappa scores all GMFCS levels included were combined and 95 % nonparametric bootstrap confidence intervals were added based on a 1000 re-samples [27, 28].

To evaluate concurrent validity the Cobb angle was used as gold standard. Area under receiver operating characteristic curves (AUC), sensitivity, specificity and predictive values were calculated. The cutoff point chosen for clinical examination was no or mild scoliosis versus moderate or severe scoliosis. We used averaged ratings for analyzing validity of the scoliometer but not for calculation of kappa values.

The AUC is a measure of the capacity of a test to classify a person correctly. In this study the AUC was used as a measure of the capacity to correctly identify scoliosis according to our definition. A value of <0.5 is not better than random, >0.7 is acceptable, >0.8 is excellent, and >0.9 is an extraordinary capability [29].

Likelihood ratio (LR) is a summary of the diagnostic accuracy of a test telling the ratio of the probability of a certain test result in individuals who do have the disease to the probability in individuals who do not. The definition of a positive LR is sensitivity/ 1-specificity. The definition of a negative LR is 1-sensitivity/ specificity. The further a LR is from 1 the greater effect it has on the probability of scoliosis, e.g. a positive LR >10 means that a positive test is good at confirming a scoliosis, while a negative LR <0.1 means that a negative test is good at ruling out a scoliosis [30]. For all statistical computing R software environment version 3.0.0 and STATA version 13.1 were used.

Ethical approval was granted by the Medical Research Ethics Committee at Lund University, Dnr 467/2013.

## Results

In total, 28 children with CP (14 boys), with a median age of 12 years (range 6–16 years) were included. All 28 children completed both the clinical and the radiographic examinations. The reassessment of the Cobb angle measurement by a pediatric orthopaedic surgeon did not result in correction of any measurements. There were nine children in GMFCS II, seven in GMFCS III, six in GMFCS IV and six in GMFCS V (Table 1).

**Table 1:** Details of all participants and distribution of scores for the 3 raters (A, B, C) for clinical examination and scoliometer measurement versus radiographic Cobb angle

Case	Age	GMFCS	Sex	Clinical examination^1^			Scoliometer^2^			Cobb-
Number				A	B	C	A	B	C	Angle^3^
1	6	IV	F	No	No	No	1	1	3	16
2	7	III	M	No	No	No	1	3	3	9
3	7	V	F	No	No	No	2	2	3	13
4	9	III	M	No	No	No	1	0	1	13
5	9	V	M	Mild	Mild	Mild	5	5	3	12
6	10	III	F	No	No	No	4	2	1	16
7	10	V	M	No	No	No	3	2	2	8
8	10	II	M	No	No	No	1	1	2	10
9	10	III	M	Mild	No	Mild	4	3	6	7
10	10	II	F	No	No	No	2	2	3	0
11	10	III	F	Moderate	Moderate	Moderate	5	6	5	23
12	10	V	M	Severe	Severe	Severe	13	18	12	37
13	11	IV	F	No	No	No	2	0	3	14
14	12	II	F	Mild	Mild	Mild	5	7	5	13
15	12	V	F	Mild	Mild	Mild	5	2	6	21
16	12	II	M	Mild	No	No	5	1	1	11
17	12	II	M	No	No	No	1	1	2	0
18	12	II	F	Mild	Mild	Mild	2	3	3	13
19	13	IV	M	Mild	Mild	Mild	4	5	5	15
20	13	II	F	No	No	No	2	2	3	13
21	13	IV	M	Severe	Severe	Severe	14	20	15	38
22	14	V	F	Mild	Mild	Mild	5	8	7	7
23	15	II	M	Mild	Mild	Mild	5	5	5	3
24	15	III	M	No	No	No	1	2	1	17
25	16	IV	F	No	No	No	1	2	2	0
26	16	IV	M	No	No	No	1	1	1	19
27	16	III	F	Moderate	Moderate	Moderate	7	10	10	9
28	16	II	F	No	Mild	No	6	6	6	11

^1^Classified as No, Mild, Moderate or Severe scoliosis

^2^Angulation of the trunk rotation in degrees

^3^Angulation of the curve on radiographs in degrees

### Clinical examination

In 25 of the 28 children there was a total interrater agreement. Among these 25 children 14 had no scoliosis, 7 mild, 2 moderate, and 2 had severe scoliosis (Table 1). The 21 children with no or mild scoliosis had an average Cobb angle of 11° (range 0–21°). The 4 children with moderate or severe scoliosis had an average Cobb angle of 27° (range 9–38°). They were all classified as GMFCS III-V, and between 10 to 16 years of age. Interrater disagreement occurred in 3 cases (cases 9, 16 and 28; Table 1). The spines were graded as no or mild scoliosis by all three raters. The average Cobb angle for the 3 children was 10° (range 7–11°). The interrater reliability of the clinical examination showed a weighted Kappa value of 0.96 (95 % CI: 0.82–1.00). The sensitivity was 75 % (95 % CI: 19.4 %–99.4 %), the specificity was 95.8 % (95 % CI: 78.9 %–99.9 %) (Table 2–3). The AUC was 0.85 (95 % CI: 0.61–1.00), the positive LR was 18 and the negative LR was 0.3. The positive predictive value was identical to the values for sensitivity, and the negative predictive value was the identical to the values for specificity (Table 3).

**Table 2:** Number of positive and negative cases of clinical spinal assessment and scoliometer measurement versus radiographic Cobb angle. (Average ratings of 3 examiners)

		Clinical assessment			Scoliometer		
		Positive	Negative	Total	Positive	Negative	Total
Cobb angle	Scoliosis ≥ 20°	3	1	4	2	2	4
	No scoliosis ≤ 20°	1	23	24	2	22	24
	Total	4	24	28	4	24	28

**Table 3:** Concurrent validity with 95 % confidence intervals (CI) of clinical spinal assessment and scoliometer measurement versus radiographic Cobb angle

	Clinical assessment vs Cobb	Scoliometer vs Cobb				
		95 % CI			95 % CI	
Sensitivity	75 %	19.4 %	99.4 %	50 %	6.8 %	93.2 %
Specificity	95.8 %	78.9 %	99.9 %	95.8 %	73 %	99 %
ROC area	0.85	0.61	1	0.71	0.42	0.99
Likelihood ratio (+)	18	2.4	133	6	1.2	31.2
Likelihood ratio (−)	0.3	0.1	1.4	0.5	0.2	1.5
Positive predictive value	75 %	19.4 %	99.4 %	50 %	6.8 %	93.2 %
Negative predictive value	95.8 %	78.9 %	99.9 %	91.7 %	73 %	99 %

### Scoliometer

In 23 of the children all three examiners measured the scoliometer angle <7°. The average scoliometer angle in these children was 3° (range 0–6°) and the average Cobb angle was 12° (range 0–23°). In 3 of the children all examiners measured the scoliometer angle above the cutoff of ≥7°. They were classified as GMFCS III, IV and V and were 10, 13 and 16 years old. The average scoliometer angle for these 3 children was 13° (range 7–20°, Table 1). Two children were measured both below and above the cutoff. In one child (Case 14; Table 1) two of the examiners measured 5° and the third examiner measured 7°. The Cobb angle was 13°. In the second child (Case 22; Table 1), one examiner measured 5° while the other two examiners measured 7° and 8° respectively. The Cobb angle was 7°. The interrater reliability of the scoliometer measurement showed a weighted kappa value of 0.86 (95 % CI: 0.64–0.92). The sensitivity was 50 % (95 % CI: 6.8 %–93.2 %), the specificity was 91.7 % (95 % CI: 73.0–99.0 %) (Table 2–3). The AUC was 0.71 (95 % CI: 0.42–0.99), the positive LR was 6 and the negative LR was 0.5 (Table 3). The positive and negative predictive values were identical to values for sensitivity and specificity (Table 3).

## Discussion

We found both the clinical examination and the scoliometer measurement to have high interrater reliability. However, the clinical examination showed a higher specificity, sensitivity and a larger area under the curve compared to the scoliometer method. For both the clinical examination and the scoliometer, the negative and positive predictive values were the same as for specificity and sensitivity. The reason for this was that the outcome table was symmetrical on the diagonal with the same number of false positives and false negatives (Table 2). The predictive value could otherwise have been negatively influenced by the low prevalence of scoliosis. The informative value and the usefulness of the test were refined for the clinical assessment by analyzing the LR, where the results were in favor for the clinical examination compared to the scoliometer measurement.

A limitation of this study was the small number of children with moderate or severe scoliosis. Based on previous psychometric studies we calculated a sample size of six children at each GMFCS-level. Everyone involved were blinded to the spinal curves of the participants prior to the examinations. A higher prevalence of scoliosis graded as moderate or severe would most likely have resulted in a narrower confidence interval for the outcome measures. This could explain the wide CI for sensitivity for both assessment methods. However, the purpose was to evaluate screening methods used to identify scoliosis in a population of children with CP and to select those in need of further investigation. As such, especially the clinical examination seems useful in a clinical setting. The prevalence of scoliosis in this study is fairly representative for children with CP. The specificity of the clinical examination was high (95.8 %) thereby reducing unnecessary referrals for radiographic examination. The radiographic examinations were performed at three radiology departments and the Cobb angle measurements were done by three radiologists. To reduce measurement error the instructions for patient positioning, x-ray imaging were standardized and all Cobb angle measurements were confirmed through a reassessment by one orthopaedic surgeon.

There was no disagreement among the assessors regarding the rating of moderate or severe scoliosis. The higher kappa values for the clinical examination may partially be explained by the narrow range of the scale (0–3).

The cutoff point for the scoliometer measure was set to 7°. When screening, a balance has to be struck in terms of not referring too many or too few for a radiographic examination and this is related to the sensitivity and the specificity of the test. Bunnell [15] studied the outcome of spinal screening with scoliometer in adolescent idiopathic scoliosis and recommended 7° as an appropriate referral criterion. To use 7° as the referral criterion reduced the prognostic referral rate from 12 % to 3 % compared to if 5° would have been the criterion for referral. When creating guidelines for assessment of adolescent idiopathic scoliosis Coelho [17] et al. concluded that if a cutoff point of 5° for scoliometer measurement was used the sensitivity would be approximately 100 % and the specificity approximately 47 %. However, if 7° was used as the cut-off instead, the sensitivity dropped to 83 % while the specificity rose to 86 %. The results from Coelho and colleagues informed the choice of cut-off point in this study. The purpose of screening for adolescent idiopathic scoliosis is to detect scoliosis in time to start bracing, and thereby reducing the need for surgery. In neuromuscular scoliosis, an additional purpose of screening is to find a progressive scoliosis in time for surgery. It is also important to identify postural asymmetries that induce a worse sitting position with poor head control, uneven weight distribution, and pelvic obliquity which could increase the development of contractures, windswept position, and hip dislocation [22].

The clinical examination and the scoliometer measurement proved somewhat difficult when examining children who had problems bending forward in sitting because of decreased flexibility of spinal muscles, short hamstrings or limited hip flexion. In one person a baclofen pump prevented the child from bending forward properly. For these individuals a moderate or severe scoliosis is often apparent when sitting in an upright position. As a consequence, when the child is unable to bend forward, the examiner is only likely to miss a mild scoliosis.

## Conclusions

In summary, this study showed an excellent interrater reliability for both the clinical spinal examination used in CPUP and for the scoliometer measurements. The sensitivity was higher for the clinical assessment compared to the scoliometer measurement, while the specificity was almost the same for both methods. There was a high validity correlated to the Cobb angle measurement. The findings should be interpreted cautiously until further research with a larger sample can verify these results. However, the clinical spinal examination used in CPUP seems to be an appropriate screening method for scoliosis in children with CP.

## Abbreviations

AUC: Area under curve
CP: Cerebral palsy
CPUP: Cerebral palsy follow up programme and quality registry
GMFCS: Gross motor function classification system
LR: Likelihood ratio
PPAS: Posture and postural ability scale
SCPE: Surveillance of cerebral palsy in Europe network
